# The Autism Spectrum Disorder and Its Possible Origins in Pregnancy

**DOI:** 10.3390/ijerph21030244

**Published:** 2024-02-20

**Authors:** Rayane M. Botelho, Ana Lúcia M. Silva, Alexandre U. Borbely

**Affiliations:** Cell Biology Laboratory, Institute of Biological and Health Sciences, Federal University of Alagoas, Maceio 57072-970, Brazil

**Keywords:** autism, ASD, pregnancy, placenta

## Abstract

Autism Spectrum Disorder (ASD) belongs to the group of neurodevelopmental disorders, and has a high prevalence, affecting 1 in 100 children according to data from the World Health Organization (WHO). To be diagnosed with ASD, the child must have persistent deficits in communication and social interactions, and restricted and repetitive patterns of behavior, interests, or activities. Despite its prevalence, the etiology of ASD is still uncertain, with multifactorial characteristics, including those associated with the gestational period, where maternal exposure to biological, chemical, or physical hazards occurs, some of which have already been proposed as causes of ASD outcomes. Since pregnancy requires a balance between the maternal–fetal binomial, the breakdown of this balance caused by such environmental hazards can lead to altered fetal neurodevelopment, including ASD. With this firmly in mind, this review aims to compile the most recent data on the gestational causes that may be associated with the development of ASD to help health professionals identify risk factors and act for the prevention and management of ASD.

## 1. Introduction

Autism Spectrum Disorder (ASD) is classified as a neurodevelopmental disorder that begins to manifest from early childhood [1]. The global average estimate shows that ASD has a prevalence of 1 in 100 children [2]. It is also known that the incidence of ASD is four times higher in males [3]. The exact cause of the predominance of ASD in boys has not yet been fully elucidated, but it is believed to be correlated with sex chromosomes and hormones [4].On the contrary, some studies justify this higher incidence of ASD in boys by the criteria currently adopted for diagnosis, which do not fully take into account the symptoms presented by females [5,6,7,8,9]. Such differences have been observed in the clinical, cognitive, and biological presentation of ASD in females, in addition to the fact that females are more socially motivated and tend to mask social difficulties [5,6,7,8,9]. Over the years, there has been an increase in the average prevalence of ASD cases worldwide, which may be due to a better understanding of the disorder and consequent diagnosis and notification, as well as shedding light on the changes surrounding exposure to environmental risk factors [10].

The diagnosis of ASD can be difficult, as there are no clinical or laboratory tests and it is necessary to evaluate the history of development and behavior [11]. It requires the presence of two main groups of symptoms related to deficits in communication and social interaction and restricted and repetitive patterns of behavior, interests, or activities [12]. The diagnosis can be made in the first few years of a child’s life. This helps to differentiate the characteristic signs of ASD from development within the expected range for their age group or other conditions that may affect child development [10]. Access to a fast and accurate diagnosis could make a difference to their lives, as it can enable the family to find specialized help and reduce the chances of possible limitations that may last into adulthood.

Despite advances in diagnostic criteria for ASD, its etiology is still debated, although it is widely accepted that it is multifactorial [13]. It is commonly accepted that abnormalities in the development of the central nervous system (CNS) contribute to the onset of ASD, such as reduced fiber density in the white matter of the brain, and atypical activation in the prefrontal cortex, superior frontal gyrus, and middle and superior temporal gyrus [14,15]. In addition to the aforementioned malformations, genetic factors have been identified as highly significant in the etiology of ASD, due to the high heritability of the disease and the association with fragile X syndrome (FXS), Down syndrome, Prader–Willi syndrome (PWS), genetic microdeletions, and single nucleotide polimorphisms and mutations [16,17,18,19,20]. This information reveals the heterogeneity of the causes that can trigger ASD and, in this context, it is necessary to shed light on the impacts of exposure to environmental factors and complications during pregnancy as possible determinants for the development of ASD [21,22].

Therefore, primary prevention involves making health professionals aware of the stressors that increase the risk of ASD, with a particular focus on counseling pregnant women, as pregnancy is a critical time for fetal development.

Clinical practice has a fundamental role in the early identification of ASD characteristics by health professionals, allowing them to assist parents in observing symptoms. In addition, understanding the etiology of ASD is essential for improving clinical care and positively impacting prenatal and childcare consultations. Therefore, this review aims to compile the most recent data on environmental exposure to certain substances in pregnancy that have been associated with the development of ASD, also helping health professionals to identify risk factors and act for the primary prevention and management of ASD.

## 2. The Influence of Environmental Hazards

The fetal period is essential for the proper development of the human brain, as it is a time when the production, migration, and differentiation of neurons and the specialization of different brain regions occur [23]. The nervous system formation events can be heavily influenced by the environment, either by biological signals from the mother or by hazardous external factors, which can contribute to behavioral dysfunction throughout life [23,24]. It is already known that the use of substances such as alcohol and other drugs, maternal stressors, and environmental factors can be detrimental to fetal neurodevelopment [25]). Therefore, it is necessary to understand how these factors can affect the development of ASD in children exposed in the prenatal period and how to avoid such exposure.

### 2.1. Air Pollution

Air pollution is a complex phenomenon characterized by a heterogeneous combination of particles, gases (such as ozone and nitrous oxide), metals, and organic contaminants, varying in size [26]. Numerous studies emphasize the significant contribution of pollution to various health issues [26,27,28]. While it is established that air pollution significantly impacts the airways and cardiovascular system, evidence indicates its negative effects on the brain due to inflammatory processes and the development of oxidative stress, contributing to the onset of central nervous system diseases [26,29].

It has been demonstrated that children exposed to pollution during the pre- and/or postnatal periods exhibit biochemical and behavioral alterations, neuroinflammatory markers, and delays in psychomotor development [29]. In utero exposure to pollutants is identified as a trigger for motor alterations and impulsive behavior, particularly in males [29]. Magen-molho and colleagues conducted a case-control study involving children born between 2007 and 2012 in Israel, who were diagnosed with ASD [30]. The results revealed a positive association between ASD and gestational exposure to air pollutants, specifically PM2.5 (particulate matter < 2.5 μm) and PM10 [30]. Statistical analysis demonstrated an increased likelihood of the development of ASD in relation to exposure to atmospheric pollutants [OR (odds ratio) per interquartile range of 1.08 (95% confidence interval: 1.01–1.15)] [30]. These findings suggest that prenatal exposure to fine particles and coarser particulate matter in the air may be associated with an elevated risk of ASD [30]. Similarly, Rahman and colleagues conducted a retrospective population-based cohort study, involving 294,937 mother–child pairs with singleton births at Kaiser Permanent Southern California (KPSC) hospitals in the USA [31]. The findings revealed a positive association between exposure to environmental pollutants and ASD. Exposure to PM2.5 in the first two trimesters showed a significant association with ASD, with a cumulative HR = 1.14 (95% confidence interval: 1.06, 1.23) [31]. Additionally, it was noted that early exposure to PM2.5 during pregnancy had a stronger association with male gender, with an HR = 1.16 (95% CI: 1.08, 1.26) [31]. These findings underscore the relevance of air quality during pregnancy as a potential risk factor for ASD development, particularly concerning the timing and gender of the fetus [31]. This concept is further corroborated by a population cohort study conducted by Yu and colleagues in Southern California, USA [32]. Over this period, 318,751 mother–child pairs were meticulously monitored through electronic medical records, leading to the diagnosis of ASD in 4559 children before the age of 5 [32]. Within this context, the researchers established a noteworthy association between exposure to air pollutants PM2.5, but not PM10, and an elevated risk of ASD development [HR = 1.07 (95% CI 1.03–1.12) per IQR = 3.73 μg/m^3^] [32].

To elucidate the mechanism by which pollutants increase the risk for ASD, Block and colleagues conducted a study using an animal model in which pregnant mothers were co-exposed to environmental pollution and stressors such as inadequate housing [33]. The results revealed an overactivation of the immune system in response to this exposure. Interestingly, lasting behavioral changes and social interaction alterations were observed exclusively in male offspring [33]. Furthermore, the study highlighted a significant decrease in microglial function and phagocytic capacity within the anterior cingulate cortex of these males [33]. These findings suggest that environmental stressors impact the formation of neural circuits in males, impairing microglial function during development [33]. The identified evidence supports the hypothesis that inflammatory processes triggered by air pollutants during the fetal developmental phase significantly contribute to the onset of ASD [26].

### 2.2. Organophosphate and Organochlorine Insecticides/Pesticides

Organochlorine and organophosphate pesticides belong to the class of toxic substances that are capable of altering neurodevelopment during the gestational period, as they can cross the placental barrier and cause alterations in fetal development [34]. Perinatal exposure to these agents causes alterations in neurotransmission and is considered a risk factor for the development of ASD [35,36]. Because of their lipophilic properties, these agents have a potent neurotoxic effect, being able to allow the sustained opening of sodium channels and interaction with gamma-aminobutyric acid (GABA) receptors, among other changes in the central nervous system [37,38].

De Felice and colleagues, using an animal model, showed that exposure during pregnancy to non-toxic levels of organophosphate insecticides, at a dose of 6 mg/kg/bw, increased oxidative stress and generated alterations in prostaglandin E2 production, which is associated with alterations in neurodevelopment and behavioral deficits [39]. These results corroborate the findings of Morales-Navas and colleagues, who also associated prenatal exposure to organophosphates and the development of ASD-like symptoms in an animal model (one subcutaneous injection of 1 mg/kg of CPF [O,O-dietil O-(3,5,6-trichloropyridin-2-yl) phosphorothioate] dissolved in DMSO [dimethyl sulfoxide], for four days) [40]. Biosca-Brull and collaborators showed in a pre-clinical model that prenatal exposure to organophosphate compounds (1 mg/kg/day dose of CPF), but not postnatal exposure, caused impairment in their social interaction, where males were the most affected [41]. In this way, they demonstrated that the moment of contact can be crucial for the development of ASD [41]. Human studies using a cohort of more than 200 pairs of mothers and children showed an association between the concentration of dimethylthiophosphate (DMTP) (1.8 μg/L in mother’s urine), a metabolite of organophosphates, and a higher risk of ASD diagnosis in girls only [OR = 1.64 (95%CI, 0.95; 2.82)] [42]. It is worth noting that the study by Philippat and collaborators was carried out with a cohort of children who were already known to have first-degree relatives diagnosed with ASD [42]. Another cohort study of more than 700 French children found that the presence of organophosphate insecticide metabolites in the urine of pregnant women, specifically chlorpyrifos, is associated with an increase in autistic traits in 11-year-old children (OR = 2.21, 95% CI = 1.32, 3.69) [36].

Exposure to organochlorine pesticides during pregnancy is associated with a reduction in cognitive development and changes in motor functions, characteristics of ASD [38]. Studies found that exposure to organochlorine compounds doubles the chance of developing ASD with intellectual disability [37,43]. Biosca-Brull and colleagues also showed that, in humans, prenatal exposure to organochlorine and organophosphate compounds increases the chance of cognitive and behavioral alterations related to ASD symptoms [35]. A study evaluating the presence of organochlorine pesticides in breast milk and their association with ASD in Norwegian children, through a prospective evaluation of a birth cohort, was able to identify that β-Hexachlorocyclohexane (β-HCH) is associated with a significantly higher risk of developing ASD in children (OR = 1.82, 95% CI: 1.20, 2.77) [44]. In this same study, the authors observed the toxicity of β-HCH in a zebrafish model and confirmed the neurotoxicity of this substance, suggesting the disruption of dopaminergic neurons as a possible mechanism underlying the onset of ASD [44]. In addition, the inhibition of acetylcholinesterase (AChE), voltage-gated sodium channels, and GABA are also mechanisms that may be acting for the development of ASD in children exposed to pesticides [45].

The mechanisms by which pesticides cause neurotoxicity and the subsequent development of psychiatric disorders are not yet fully understood. Nonetheless, López-Merino and colleagues observed in animal models that exposure to organochlorine and organophosphate pesticides in the perinatal period induces chronic alterations in the MAPK/ERK signaling pathway, which is essential for regulating synaptic plasticity [46]. Furthermore, they demonstrated that metabotropic glutamate receptor-dependent long-term depression (mGluR-LTD) in the hippocampus was impaired after exposure to these pesticides, potentially causing the cognitive and behavioral deficits observed in ASD [46].

On the contrary, a recent systematic review demonstrated the absence of an association between prenatal exposure to endocrine disruptors, such as pesticides, and the development of ASD [47]. However, this emphasizes that it may be due to methodological differences in the selected studies, and that the risk of pesticide exposure in the development of ASD cannot be ruled out [47].

### 2.3. Microplastics and Additives

Global plastic production has increased exponentially since the 1950s [48]. Although necessary in modern lives, only 9% of all plastic waste is recycled, while 22% pollutes the environment as macro- and microplastic waste [48]. Macroplastic pollution is degraded by physical elements (e.g., sun, heat, wind) and chemical agents (e.g., acid, salt, chlorine) into microplastics (MP, 1 µm to 5 mm) [49]. These microplastics accumulate in the environment over time [27]. Recently, there have been reports of MP accumulation in several organs and fluids, including placentas and breast milk [50,51]. Nonetheless, the impact of MP accumulation in human tissues and the effect on population health are still unclear [51].

MPs differ in terms of size, color, type, and polymer composition [52]. A study carried out by Chen and collaborators showed that polystyrene (PS) microplastics, using a zebrafish model, did not cause apparent toxicity in embryonic development but was able to generate accumulations in the head and nervous tube of zebrafish embryos, and concluded that PS found in the oceans can possibly generate toxicity for neurodevelopment; however, it did not aim to evaluate behavior similar to ASD [52]. A study evaluating the polyethylene (PE) polymer, on the other hand, proposes that MPs trigger ASD-like symptoms in mice offspring when their mothers were fed with PE and it was observed that MPs showed bio-accumulation in the brain after the digestion process [53]. In addition, the offspring showed a reduction in social interactions and in spatial memory, hesitation, decreased interest in exploring new environments, and an increase in repetitive and compulsive behaviors [53].

Additionally, MPs are usually not alone, carrying additives such as plasticizers, heavy metals, dyes, and others, which are known to be endocrine disruptors, some being associated with ASD [54]. Plasticizers such as phthalates are highly used in the industry to manufacture plastics, hospital products, cosmetics, and toys, and can cross the placenta and reach fetal tissues, in addition to impacting brain development [28,55,56]. Moreover, exposure to phthalates in various forms and concentrations can lead to hyperactivity, impulsivity, and inattention in children diagnosed with ASD [57]. In a study conducted by Kardas and collaborators, phthalate levels were elevated in children with ASD [56]. Interestingly, Oulhote and colleagues, when evaluating the urine of pregnant women in the first trimester, identified that higher concentrations of phthalate metabolites in urine during the gestational period were correlated with high scores of autistic traits in their children, specifically in boys aged between 3 and 4 years old [58]. It is interesting to note that another similar study, performing analyses of the urine phthalate levels of pregnant women (11–18 weeks of pregnancy), showed an increased concentration of phthalate metabolites, not only in pregnant women but also in the urine of male neonates, although not in female neonates [59]. These male neonates had increased difficulty with social skills, such as emotional response, anxiety, depression, somatic complaints, social isolation, and behavioral problems as they were growing and being followed by the study [59].

To understand how phthalates can be responsible for the development of ASD, Li and colleagues, using an animal model, observed an increase in the expression of components related to the mTOR pathway and the inhibition of autophagy in the brains of offspring exposed to phthalates during the perinatal period [60]. These alterations may be responsible for the development of symptoms resembling the autistic spectrum in these animals [60].

Another plasticizer, bisphenol A (BPA), is widely used in the manufacture of plastic and epoxy resins and is known to be an endocrine-disrupting chemical [4]. Animal studies have shown that the presence of BPA during pregnancy has a significant impact on the nervous system [61]. It was observed that, after exposure to this plasticizer during the pregnancy of rats and up to three days after birth, the offspring showed a reduction in the number of neurons, as well as a decrease in the astrocytic population with reduced dendritic spines [61]. In the same study, behavioral changes similar to anxiety could be identified in offspring exposed to BPA [61]. Another study with fetal exposure to BPA showed increased expression of transcription factors related to the development of ASD, such as the androgen receptor (AR), and against decapentaplegic homolog 4 (SMAD4) in male, but not in female, offspring [4]. The exact cause and mechanisms underlying the male bias of ASD are still unclear, but there is evidence that sex chromosomes and hormones are also involved in ASD susceptibility [4].

Furthermore, Hansen and collaborators carried out a prospective study to assess how intrauterine exposure to BPA could be associated with ASD symptoms, quantifying BPA in the urine of women at 28 weeks’ gestation [62]. As a result, they found that the children of mothers with higher levels of BPA had a potentially higher risk of developing ASD in children at the age of five [62].

The presence of MPs in the placenta was only recently discovered, and we do not know for sure how they impact placental and embryonic development. It is essential to assess the behavior of these MPs in the placenta since they could promote neurodevelopmental changes.

### 2.4. Heavy Metals

It has been known for some time that the exposure to heavy metals during pregnancy can negatively impact fetal development, with changes in neurodevelopment and behavior [63]. Toxic metals, such as arsenic (As), cadmium (Cd), lead (Pb), and mercury (Hg) have been associated with the onset of ASD due to dysregulation in the neurotransmissions and changes in the frontal and subcortical regions of the brain [64,65,66,67].

In search of understanding how exposure to metals could affect the neurodevelopment of children, Freire and collaborators used the placenta as a biological exposure matrix [68]. Metal levels were measured in randomly selected placental tissues. Their levels were associated with decreases in global and verbal executive functions and quantitative skills (limit of detection was 0.0038 ng/g for As, 0.23494 ng/g for Cd, 0.016 ng/g for Hg, 0.94 ng/g for Mn [manganese] e 6.50 ng/g for Pb) [68]. Elevated placental Hg was associated with lower verbal function scores of the children, for example (OR = 2.43, 95% CI = 1.07, 5.52) [68]. These results suggest that prenatal exposure to these metals may be risk factors for cognitive and motor impairments in children—signs that may be present in ASD [68].

Exposure to heavy metals during critical stages of fetal development increases not only the risk but also the severity of ASD [65]. A systematic review with a meta-analysis carried out by Ding and collaborators identified higher levels of Cd (SMD = 0.17, 95%CI (−0.18, 0.51), *p* > 0.05], Pb [SMD = 1.16, 95%CI (0.76, 1.55), *p* < 0.001], Hg [SMD = 0.74, 95%CI (0.44, 1.03), *p* < 0.001], and As [SMD = 1.40, 95%CI (0.75, 2.05), *p* < 0.001] in children with ASD, potentially coming from maternal exposure in pregnancy [69]. Corroborating these findings, Wegmann and collaborators measured metal levels in the umbilical cords of newborns and in the plasma of these same children 5 years later [70]. Higher levels of aluminum (Al) were found in umbilical cord blood in children with ASD compared to the control group [70]. Al is associated with the severity of behavioral symptoms of ASD, being an important neurotoxic and neuroinflammatory agent [70]. In 5-year-old children with ASD, Hg levels were elevated when compared to the control group, which has already been demonstrated by previous studies [70]. It was also found that Zinc (Zn) levels in the umbilical cord blood of the control group were higher than in children with ASD, which may suggest that the population with ASD is exposed to fewer nutritional options containing this microelement [70]. Decreased Zn levels have already been associated with ASD as a risk factor [71].

### 2.5. Medications

Various drugs used during pregnancy have been associated with the development of ASD [28]. These include valproic acid, thalidomide, selective serotonin reuptake inhibitors (SSRIs), and acetaminophen (paracetamol) [28].

Valproic acid (VPA), used as an anticonvulsant agent and mood stabilizer, presents a significant risk when used during pregnancy, especially in the first trimester [72]. This drug increases the probability of fetal malformations, neural tube defects, delayed neurodevelopment, and cognitive changes, which are linked with the development of ASD [72,73]. An experimental study carried out by Zhao and collaborators evaluated molecular, cellular, and behavioral changes in cynomolgus monkeys (Macaca fascicularis) exposed to VPA (200 or 300 mg/kg) in utero. This concentration is equivalent to about 10 times the human therapeutic dose of 20–30 mg/kg/day [74]. The study observed behavioral changes, including pronounced stereotypies and defects in social interaction, in animals exposed to the drug in utero; additionally, defects in neurogenesis and altered levels of gene expression in the brain were identified [74]. According to a study conducted by Rasalam and colleagues, children exposed to valproic acid had a prevalence of ASD of 8.9% [75].

Currently, in vitro and in vivo studies have strengthened the association between the use of valproic acid and the development of ASD [76,77]. A study conducted by Meng and colleagues, using experiments with human forebrain organoids exposed to valproate (1 mM, clinically relevant concentration), demonstrated an overexpression of several genes associated with the development of autism, such as CAMK4, CLCN4, DPP10, GABRB3, KCNB1, PRKCB, SCN1A, and SLC24A2 [76]. In addition, valproate affected genes related to neural development, synaptic transmission, oxytocin signaling, and calcium and potassium signaling pathways, all of which are implicated in ASD [76]. An in vivo study evaluating the intrauterine exposure of mice to 0.4% of valproic acid identified alterations in social interaction and cognitive functions, hypersensitivity to heat and pain stimuli in offspring exposed during gestation, behaviors characteristic of ASD [77]. In this context, several studies have used the animal model of autism induced by valproic acid to study changes related to ASD, with the administration of VPA ranging from 500/600 mg/kg [78,79,80].

Moreover, Fereshetyan and collaborators, employing an animal model of prenatal and postnatal exposure to valproate, revealed distinct cellular alterations in the prefrontal cortex, amygdala, and cerebellum when exposed prenatally [81]. Interestingly, these changes were not observed when the exposure occurred after delivery [81]. Nonetheless, both prenatal and postnatal exposures led to behavioral and social interaction alterations [81]. Ishihara and colleagues, also employing an animal model, demonstrated that valproate induces microglial activation and neural circuit dysfunction in the hippocampus [82]. This effect is accompanied by the overexpression of the chemokine CCL3, contributing to neurocognitive alterations believed to be stimulated by these modifications [82]. This shows the strong involvement of the use of this drug during pregnancy with the appearance of ASD-like phenotypes.

Thalidomide, used to treat hanseniasis and multiple myeloma, has well-established teratogenic effects when used during pregnancy, including limb reduction (phocomelia) and cardiovascular and ocular abnormalities, and its use is prohibited in pregnant women [28,83]. Experiments conducted by Narita and colleagues on rats showed that exposure to 500 mg/kg, especially in early pregnancy, led to the development of ASD characteristics [84]. Thalidomide may increase the risk of autism by altering the expression of genes associated with neurotransmission, as indicated by Zieminska and collaborators [85].

The SSRIs, often prescribed to treat depression, have been associated with obstetric and neonatal changes, with a potential impact on children’s neurobehavioral development [86]. These drugs cross the placental barrier, representing a significant risk factor for ASD [86,87]. Using an animal model of prenatal SSRI exposure, Bhat and colleagues identified that this medication induces biochemical changes in the brains of rats [88]. This includes lipid peroxidation, an increase in inflammatory cytokines, and apoptotic proteins such as Interferon-gamma (IFN-γ) and caspase-3, respectively [88]. Additionally, the use of SSRI during pregnancy resulted in a reduction of antioxidant molecules in the brain, such as glutathione (GSH), glutathione S-transferases (GST), and catalase [88]. These alterations may represent potential mechanisms through which SSRI use during pregnancy increases the risk of ASD.

Studies conducted by Bérard and colleagues, with mothers exposed to SSRIs during the second and third trimesters of pregnancy, showed an increased risk of developing ASD, ranging from 1.87 to 8.36 among the study participants [89]. However, the exact gestational period of exposure related to the onset of ASD is not yet fully understood [28]. An antagonistic result was obtained from a case-control study carried out in the United States with children born between 2003 and 2011, in which it was found that, among mothers with psychiatric disorders, the use of SSRIs was not related to the presence of ASD in their children [90]. Corroborating this finding, a study by Brennan and collaborators showed that no association was found between exposure to antidepressants during pregnancy and the appearance of traits or a diagnosis of ASD, based on a retrospective study with multiple cohorts [91].

Acetaminophen (paracetamol), widely used by pregnant women as an antipyretic and analgesic, is also associated with alterations in neurodevelopment [92]. In order to assess the possible effects of using paracetamol during pregnancy, Klein and colleagues carried out an experimental study using rats exposed to this medication (350 mg/kg/day) during pregnancy and were able to observe that the female pups developed stereotyped behavior, in addition to inducing signs of hyperactivity in both sexes, these signs being similar to those presented during ASD [93]. Looking at studies with humans, a Danish study that followed more than 64,000 children and mothers for 12 years revealed that 1.6% of the children involved were diagnosed with ASD [94]. In addition, research conducted by Avella-Garcia and colleagues showed that prenatal exposure to this drug increases ASD symptoms in male children, affecting attention and generating hyperactive behaviors [92]. More recently, a prospective cohort study of more than 900 mother–child pairs used umbilical cord blood to verify the relationship between acetaminophen metabolites and the development of ASD [95]. The study found that biomarkers of acetaminophen metabolism are dose-dependently correlated with an increased risk of developing ASD in childhood (ORs: 1.6 to 4.1) [95]. Baker and colleagues, using an animal model, observed that acetaminophen treatment during the prenatal period induced changes in behavior and gene expression in the prefrontal cortex related to glutathione and cytochrome P450, DNA damage, and immune and endocrine systems in the puppies [96]. These alterations were primarily present in males [96].

### 2.6. Alcohol Consumption

Maternal consumption of alcoholic drinks during pregnancy has a significant impact on children’s neurodevelopment, intellectual capacity, and learning ability [97,98]. Often, this consumption can lead to the development of Fetal Alcohol Spectrum Disorder (FASD), which is characterized by dysmorphic facial features, fetal growth restriction, and restricted brain growth, resulting in neurobehavioral impairment throughout life [97,98,99,100]. Although they share similarities, FASD and ASD are distinct entities. FASD is a group of conditions associated with prenatal exposure to alcohol, characterized by somatic and neuropsychological alterations, while ASD is a neurobehavioral syndrome [101].

Gallagher and colleagues, through a retrospective study, classified alcohol consumption based on the number of units of beverages consumed, including half a liter of beer, a glass of wine, or a single measure of alcoholic beverage [99]. Consumption is considered light when one to two units are consumed per week or at any time during pregnancy, moderate when three to tsix units are consumed per week, or three to five units at any time during pregnancy, and heavy when seven or more units are consumed per week or six or more units are consumed at any time during pregnancy [99]. In this context, the authors found no association between alcohol consumption in pregnancy and the development of ASD [99]. Similarly, Eliasen and colleagues found no positive relationship between alcohol consumption during pregnancy and the development of ASD in children born to mothers who consumed alcohol during pregnancy [102]. However, a study conducted by Stevens and colleagues that evaluated children with FASD identified notable similarities in social and communicative behavior between these children and those with ASD [97]. Sowel and collaborators demonstrated that alcohol use during pregnancy can trigger an imbalance between pro- and anti-inflammatory cytokines in pregnant women, which in turn can result in alterations in fetal neurodevelopment [103]. In this study, a significant increase in pro-inflammatory cytokines, such as TNF-a and IL-6, was observed in relation to IL-10 levels, and this discrepancy correlated negatively with the children’s psychomotor and mental development at 12 months of age [103].

The divergent correlations between ASD and alcohol exposure during pregnancy highlight the need for more research and in-depth studies on this subject.

### 2.7. Hyperglycemia—Diabetes Mellitus

Diabetes mellitus (DM) is a term used to designate metabolic disorders that present with chronic hyperglycemia [104]. It can be classified as type I, when there is destruction of pancreatic β cells, leading to insufficient insulin production; type II, when there is resistance to insulin produced by the body, which may be at insufficient levels; and gestational diabetes, when this metabolic disorder appears for the first time during pregnancy [104].

Gestational Diabetes Mellitus (GDM) is a chronic condition prevalent during pregnancy and which affects a large number of pregnant women worldwide [104]. In addition to elevated glucose levels, this disease is accompanied by an imbalance in the mother’s inflammatory response, with an increase in the production of pro-inflammatory cytokines to the detriment of anti-inflammatory ones [105,106]. The underlying pathophysiology of GDM involves modifications in the β-cells of the pancreas, caused by excess human placental lactogen hormone (hPL) [107]. This leads to the development of insulin resistance, resulting in hyperglycemia and excessive glucose supply to the fetus [107]. The high maternal glycemic index negatively impacts fetal brain development, and is associated with the emergence of disorders in growth and fine and gross motor development, as well as learning difficulties, all factors present in ASD [83,108].

A retrospective cohort study carried out in Taiwan, with 877,233 children born between 2004 and 2008, showed a correlation between GDM and a higher risk of ASD, attention deficit hyperactivity disorder, and developmental delay [109]. In addition, they investigated the relationship between DM types I and II and the development of ASD, concluding that DM II was associated with a higher risk of ASD, among other neurodevelopmental disorders [109]. Liu and his colleagues, through a case study, also confirmed this positive link between GDM and the risk of developing ASD, highlighting that male children of mothers with GDM were more likely to develop ASD [110]. Persson and colleagues, in a cohort study involving 1.4 million Swedish children, also identified that a previous diagnosis of type I DM before pregnancy is a risk factor for ASD, increasing the chance of developing ASD by approximately 1.4 times [111].

In an attempt to understand the mechanism by which gestational hyperglycemia may induce ASD, Yu and colleagues, using a maternal diabetes animal model, concluded that the presence of hyperglycemia during pregnancy led to the suppression of Retinoic Acid-Related Orphan Receptor Alpha (RORA) in offspring, which is associated with autism development [112]. Furthermore, gestational hyperglycemia may result in the suppression of Superoxide Dismutase (SOD) and persistent oxidative stress in the nervous system of the offspring, leading to behaviors resembling the autism spectrum [113].

### 2.8. Maternal Obesity

Maternal obesity has become an increasingly evident issue worldwide, often associated with an increase in maternal and neonatal mortality and morbidity [114]. Given the high prevalence of obesity, it is important to understand its impact during pregnancy, as this condition involves alterations in lipid metabolism and the presence of chronic inflammation, situations that can affect the intrauterine fetal development programming [114,115].

It is worth noting the significant involvement of cholesterol and essential fatty acids in fetal neurodevelopment and how inflammatory conditions are linked to disruptions in children’s neurological development [115,116]. It is interesting to assess the impacts of maternal obesity on the etiology of ASD. A meta-analysis by Zhang and colleagues observed the impact of maternal overweight/obesity on the mental health of children [117]. They highlighted that maternal obesity before pregnancy increased the risk of ASD [OR 1.37 (95% CI 1.22, 1.55)], as well as other neurodevelopmental alterations [117]. In this scenario, Matias and colleagues, through a case-control study across various locations in the USA, observed that maternal obesity before pregnancy (BMI 35.0 to ≥40 kg/m^2^) was associated with approximately twice the probability of ASD development (Adjusted OR [AOR] = 1.87, 95% CI: 1.40–2.51) [118]. Additionally, when looking at weight gain during pregnancy, they found a greater association with the development of ASD in males (AOR = 1.47, 95% CI: 1.15–1.88) [118]. Supporting this finding, another case-control study conducted with children born in California, USA, utilizing multivariate logistic regression, identified that maternal obesity was also associated with the diagnosis of childhood ASD (OR = 1.51, 95% CI: 1.07–2.13) [116]. Similar results were also found by Carter and colleagues in their retrospective, population-based cohort study, which included 308,536 mother–child pairs [119]. It revealed that maternal obesity had a higher association with the probability of ASD onset with gastrointestinal disorders (OR = 1.37, 95% CI: 1.22–1.54) [119].

As previously mentioned, obesity induces a dyslipidemic state. In order to determine the association between maternal postpartum lipid profile and the risk of ASD in children, Park and colleagues conducted a case-control study involving a total of 7939 mother–child pairs [115]. A higher association was observed between mothers with elevated BMI (Body Mass Index) and children diagnosed with ASD [115]. Interestingly, postpartum maternal levels of total cholesterol (*p* = 0.03) and LDL (*p* = 0.01) were significantly lower in mothers with children with ASD than in neurotypical controls [115].

In a prospective birth cohort study analyzing data from 756 mother–baby pairs (86 with ASD) from the Boston Birth Cohort, Panjwani and colleagues identified that low maternal postpartum high-density lipoprotein cholesterol (HDL-C) was associated with an increased risk of ASD [120]. Affirming the previously demonstrated results, Lin and colleagues, through the analysis of data collected from medical records of over 300 children diagnosed with ASD, found that obesity, accompanied by reduced cholesterol levels, are significant factors associated with an ASD diagnosis [121].

Hasegawa and colleagues conducted a longitudinal investigation in obese and healthy non-human primates, as well as their offspring, aiming to better comprehend the mechanisms that correlate maternal obesity with the metabolism and cognitive processes in the offspring [122]. The results revealed that maternal obesity was associated with elevated levels of inflammation and significant alterations in the metabolites linked to energy metabolism in both maternal plasma and urine samples, as well as in the placental tissue [122]. Furthermore, the offspring born from obese mothers exhibited behavioral and emotional changes, along with differences in the phosphorylation of enzymes in the mechanistic target of mTOR signaling pathway in the brain, which is critical for its structural and functional development [122]. These findings underscore the complexity of interactions between maternal obesity and neurological development, providing valuable insights into understanding the risks associated with ASD.

Moreover, it is crucial to highlight the adverse impact of the inflammatory process during gestational obesity, as well as to recognize the significant role played by lipids in the etiology of ASD. It is relevant to emphasize that both maternal lipid levels and obesity are potentially modifiable factors, and interventions targeted at these aspects can play a crucial role in preventing alterations such as ASD.

### 2.9. Vitamin D Deficiency

In addition to the great influence of vitamin D on the skeletal system, its impact on the development of brain functions is also well established, being fundamental for neurotrophic actions, maturation, differentiation, and neuronal growth [123]. Deficiency of this vitamin during pregnancy can cause comorbidities such as premature birth, preeclampsia, and gestational diabetes [124,125]. In addition, studies have associated vitamin D deficiency during pregnancy with neurobehavioral disorders such as schizophrenia and ASD [124,125]. Epidemiological data suggest that the development of ASD is common in areas where there is a reduction in the incidence of UVB light, which is responsible for causing the production of vitamin D by the skin, corroborating the theory that vitamin D deficiency is associated with ASD [124,125].

A case-control study carried out in Southern California between 2000 and 2003 found no association between the increased probability of ASD with maternal vitamin D deficiency, nor a protective effect of increased vitamin D levels in general [126]. Furthermore, Madley-Dowd and colleagues, using data from a UK-based cohort study involving 62 patients diagnosed with ASD between 1991 and 1992, where the pregnant woman’s vitamin D levels were recorded during prenatal care, found no evidence linking maternal vitamin D levels to a risk of developing ASD [127]. However, the study population is a limitation that may have interfered with the results of the analysis [127].

On the other hand, a prospective study was carried out using vitamin D supplementation in pregnant women who already had children diagnosed with ASD in previous pregnancies, prescribing 5000 IU of D3 per day during pregnancy [128]. As a result, they obtained a reduction in the rate of ASD recurrence among siblings, which may be due to supplementation during pregnancy [128]. However, it is important to note that this is a preliminary study [128]. Moreover, a case-control study in Finland, with vitamin D evaluation in first and second trimesters of pregnancies from 1987 and 2004, and children diagnosed with ASD and followed until 2015 showed that vitamin D insufficiency (<49.9 nmol/L) during pregnancy increased the risk of ASD [129]. Lee and colleagues, through a cohort study in Stockholm, showed that maternal vitamin D insufficiency (25–<50 nmol/L) at approximately the 11th week of gestation was correlated with a 1.58 increased risk of ASD [130].

One possible mechanism by which vitamin D deficiency increases the risk of ASD is by endocrine disruption. Using an animal model of vitamin D deficiency, Ali and collaborators demonstrated an increase in testosterone levels in both mothers and male embryos, the latter being a risk factor for ASD [131]. Additionally, they observed a significant rise in testosterone in the brains of male embryos, while the enzyme responsible for its elimination, aromatase, was reduced [131]. These findings suggest a potential association between vitamin D deficiency, hormonal changes, and the risk of ASD development [131].

Altogether, these studies suggest that lower concentrations of vitamin D in early pregnancy may be associated with an increased risk of neurodevelopmental disorders, including ASD.

### 2.10. Maternal Microbiota Dysfunction

The relationship between the gastrointestinal tract and the nervous system has attracted attention, particularly the impact of the gut microbiota on the pathophysiology of various neurological and psychiatric disorders [132]. Metabolites derived from the gut microbiota have been shown to have a direct relationship with the enteric nervous system, as well as being capable of indirect activation [133]. When the blood–brain barrier (BBB) is intact, its main effect is protection during the critical period of fetal brain development [132]. However, several different metabolites are able to cross the BBB, such as certain amino acids, carnitine, phenolic derivatives, and products of carbohydrate metabolism [133].

Given the possible involvement of the microbiota in the development of nervous system disorders, some authors have shed light on the relationship between the maternal microbiota and the emergence of neurobehavioral alterations [134,135]. A meta-analysis of experimental studies emphasized the possible effects of the maternal microbiota on the neurological and behavioral development of the fetus, observing that the disruption of the maternal microbiota negatively affects behavioral parameters in the offspring, such as sociability and obsessive-compulsive behavior [134]. In this context, knowing that the intestinal microbiota can be modulated by diet, Afroz and collaborators performed an experimental study using a mouse model of parental high salt diet (HSD), where it was observed that the dietary pattern adopted altered the maternal intestinal microbiota, which was shared with the offspring, demonstrating that maternal dysbiosis directly influences the composition of the fetal microbiota [135]. Interestingly, it was found that the offspring developed behaviors similar to ASD due to changes in the microbiota [135].

In addition to diet, another factor associated with alteration of the gut microbiota is the use of antibiotics [136]. In this scenario, Morel and collaborators conducted a study to evaluate the disruption of the maternal intestinal microbiota in mice during a critical perinatal window by the use of ampicillin and the possible neurobehavioral effects associated with ASD in the offspring [136]. As in the previous study, they identified changes in several species of gut bacteria, such as *Lactobacillus murinus* and *Parabacteroides goldsteinii*, in the offspring [136]. They observed that the offspring, especially the males, showed an altered pattern of ultrasonic communication, reduced social motivation and social interaction, as well as anxiety-like behavior [136]. In this context, a population-based study in Sweden examined the association between antibiotic use during pregnancy and ASD diagnosis and found that maternal antibiotic use was associated with a higher risk of childhood ASD [(OR) = 1.16, (95% CI) 1.09–1.23] [137]. It is suggested that changes in the maternal microbiota caused by antibiotic use may play a role in the development of this disorder [136,137].

To better understand the impact of maternal microbiota, Cristiano and collaborators evaluated the effect of maternal treatment of BTBR mice with the intestinal microbial metabolite butyrate (BUT), a short-chain fatty acid (SCFA) produced in the colon by bacterial fermentation of dietary fiber and resistant starch [138]. In this study, the authors found that BUT attenuated ASD-like behaviors in offspring and reduced morphological and functional alterations of the cerebellar cortex in offspring [138]. This demonstrates how modulation of the maternal gut microbiota can have beneficial effects in preventing ASD-like behaviors.

Experimental studies using a preclinical mouse model highlight that maternal intestinal commensal bacteria can induce Th17 cells and interleukin 17 (IL-17A), which could potentially increase the risk of neurodevelopmental disorders in the children of pregnant mothers exposed to immune system activation due to infection or autoinflammatory syndromes [139,140]. In addition, Morel and colleagues found that the behavioral phenotype found in male offspring of mice with disturbed gut microbiota was associated with decreased gene expression of the oxytocin receptor (OXTR) and identified a reduction in several tight junction proteins in the prefrontal cortex, a region associated with the process of regulating social and emotional behaviors [136].

It should be emphasized that the evaluation of maternal microbiota and the etiology of ASD is still an emerging topic and more human studies are needed to better define the relationship between the two factors.

## 3. The Influence of Gestational Diseases

### 3.1. Preeclampsia

Preeclampsia is a multisystem disorder that affects 3–5% of all pregnancies and is related to high rates of maternal and fetal morbidity and mortality, accounting for around 63,000 maternal deaths per year [141,142,143]. The main symptoms related to this syndrome are hypertension and endothelial dysfunction, and it can be associated with proteinuria and organ damage, such as liver, kidney, brain, and placenta [143,144]. The pathophysiology of preeclampsia is complex, but it is known that abnormal placentation is a key factor in its development [145,146]. The defective invasion of the uterine spiral arteries by extravillous cytotrophoblast cells is the main cause of the development of the most severe forms [145,146]. Due to this defect in arterial remodeling, there is an increase in vascular resistance, which can result in placental ischemia due to hypoperfusion and oxidative stress, as well as a reduction in the nutritional supply to the fetus [145,146]. Placental ischemia and insufficiency are related to various fetal alterations, such as reduced fetal oxygenation, intrauterine growth restriction, and abnormal neurodevelopment, among other complications [144,145,146].

A retrospective population study carried out in Sweden, with 1,645,455 neonates born between 2000 and 2016, compared the rate of ASD between children exposed and not exposed to preeclampsia [147]. The results indicated higher rates of ASD in children exposed to preeclampsia, suggesting the importance of defects in placental formation in the etiology of ASD [147]. Moreover, previous studies have already established a link between preeclampsia and the development of ASD [144,148,149,150]. In order to understand the mechanism underlying the relationship between preeclampsia and ASD, Liu and colleagues carried out a preclinical study using an animal model of preeclampsia [151]. The results showed that the offspring exhibited behavioral and neurodevelopmental symptoms related to ASD, with an unbalance of inflammatory markers produced by TNFα/NFκB signaling axis [151]. These findings suggest that the imbalance in the production of pro-inflammatory factors during the preeclamptic pregnancy may play a role in the development of ASD [151].

### 3.2. Viral Infections

The occurrence of viral infections during pregnancy can lead to various complications, such as miscarriage, congenital viral syndromes, and impaired fetal neurodevelopment [152,153]. These situations result from the breakdown of the immune balance that is essential for the correct development of a pregnancy [152,153]. In a population-based study involving Danish children, Atladóttir and colleagues identified a significant association between viral infections in the first trimester of pregnancy and an increased risk of ASD in their children, suggesting that early viral infection may be a risk factor for ASD [152].

Among the viruses that cause damage to the fetus is the rubella virus, responsible for Congenital Rubella Syndrome (CRS) [154]. Children with CRS have characteristics similar to ASD, such as hyperactivity, reduced cerebral perfusion in comparable areas, increased muscle tone, and a predisposition to developing type 1 diabetes [154]. These similarities support the hypothesis that the rubella virus is involved in ASD [154]. In addition to rubella, the relationship between cytomegalovirus (CMV) and ASD has been studied since the 1980s [155]. Maeyama and collaborators, in a literature review, observed a high prevalence of congenital CMV infection in children with ASD [155]. Moreover, Slawinski and colleagues found an association between CMV infection and ASD [156]. Another viral infection that is known to cross the placental barrier is the ZIKA viral infection [157]. The ZIKA virus is also linked to the development of neonatal syndromes, with microcephaly being the main alteration identified [157]. In addition to microcephaly, children affected by the ZIKA virus congenital syndrome can present alterations in neurodevelopment, and language difficulties [158]. The relationship between maternal ZIKA virus infection and the development of ASD was first investigated by Santi and collaborators in a case study [159]. The only disturbance during pregnancy was the mother’s infection with the ZIKA virus [159]. The neonate was born without microcephaly and was diagnosed with ASD at the age of 3, according to clinical tests and confirmed by DSM-V [159].

### 3.3. Bacterial Infections

During pregnancy, bacterial infections can trigger inflammation of the placenta and fetal membranes, resulting in placentitis, deciduitis, and chorioamnionitis [160]. The inflammation of these membranes is largely associated with the rise of bacteria through the vaginal canal into the uterus [160]. Common pathological microorganisms in intrauterine infections include *Ureaplasma urealyticum*, *Chlamydia trachomatis*, *Neisseria gonorrhoea*, *Mycoplasma hominis*, group B streptococcus, and *Trichomonas vaginalis* [161]. In chorioamnionitis, in addition to these, there is the possible presence of anaerobic Gram-negative organisms such as *Gardnerella vaginalis*, *Bacteroides* spp., *Staphylococcus aureus,* and *Mycoplasma* ssp. [161]. Viruses and fungi are also associated with the development of chorioamnionitis, such as CMV, enterovirus, *human papillomavirus* (HPV), *Zika virus* and other arboviruses, and *Candida* spp. [162].

The exacerbated inflammatory response triggered by the bacterial infection has the potential to cause brain damage in the developing fetus, resulting in changes in cognitive and psychological functions in children, such as cerebral palsy, schizophrenia, and ASD [160,163,164]. A population-based case-control study involving 4184 children diagnosed with ASD highlighted the potential impact of bacterial infections in the prenatal period, especially during the third trimester, by establishing an association between the presence of infection and the development of ASD [165]. Similar research conducted by Zerbo and colleagues concluded that multiple infections, especially those caused by bacteria, are related to an increased risk of ASD (ORadj = 1.36, 95% CI 1.05–1.78) [166].

In a preclinical model of maternal immune activation with lipopolysaccharide (LPS), Dutra and collaborators found that the offspring showed behaviors characteristic of ASD, such as changes in social interaction and stereotyped movements [167]. Additionally, in an animal model of GBS-induced chorioamnionitis, Bergeron and colleagues observed that only male pups developed deficits in motor behavior and social alterations [168]. Elevated levels of IL-1β, TNF-α, and polymorphonuclear cell infiltrates were predominantly present in males, correlating with the higher incidence of ASD in males [169]. Considering that IL-1β is a characteristic of the inflammatory response induced by GBS, Ayash and collaborators demonstrated in vivo that blocking IL-1β in GBS-induced chorioamnionitis at the end of gestation can result in a reduction in GBS-induced placental and fetal inflammation without aggravating the infection, indicating a possible prevention of neurobehavioral complications related to this cytokine [170].

## 4. Conclusions/Future Directions

**Figure 1:**
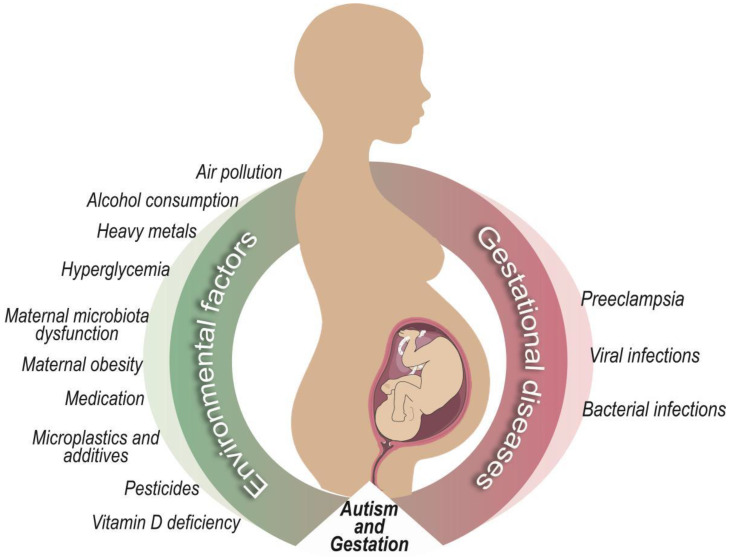
Multifactorial elements involved in risk for ASD development during pregnancy.

ASD stands out as one of the most prevalent neurodevelopmental disorders, revealing a significant increase in its incidence over the years. Understanding its causes is crucial to directing future prevention and mitigation measures, a challenge that involves health professionals and others. Faced with a scenario in which the causes of ASD are widely debated, there is a consensus that the phenomenon may be multifactorial (Figure 1).

Although the role of genetics is undeniable in many ASD cases, herein the close link between external factors and pregnancy were compiled, highlighting their significant influence on fetal development, especially on the nervous system. It is essential to understand the main risk factors associated with the environment and pregnancy, as they are modifiable and can therefore be effective targets for preventing ASD. The intersection between environment, pregnancy, and risk factors has become a fertile field for research, promising not only in-depth understanding but also effective strategies to mitigate the incidence of ASD.

## Data Availability

Not applicable.
